# Tracing the evolution of the plant meiotic molecular machinery

**DOI:** 10.1007/s00497-022-00456-1

**Published:** 2023-01-16

**Authors:** Gokilavani Thangavel, Paulo G. Hofstatter, Raphaël Mercier, André Marques

**Affiliations:** 1 Department of Chromosome Biology, Max Planck Institute for Plant Breeding Research, Carl-von-Linné-Weg 10, 50829 Cologne, Germany; 2 University of Sao Paulo, São Paulo, Brazil

**Keywords:** Meiotic proteins, Homology search, Phylogeny, Plant, Conservation, SPO11 duplication

## Abstract

Meiosis is a highly conserved specialised cell division in sexual life cycles of eukaryotes, forming the base of gene reshuffling, biological diversity and evolution. Understanding meiotic machinery across different plant lineages is inevitable to understand the lineage-specific evolution of meiosis. Functional and cytogenetic studies of meiotic proteins from all plant lineage representatives are nearly impossible. So, we took advantage of the genomics revolution to search for core meiotic proteins in accumulating plant genomes by the highly sensitive homology search approaches, PSI-BLAST, HMMER and CLANS. We could find that most of the meiotic proteins are conserved in most of the lineages. Exceptionally, *Arabidopsis thaliana* ASY4, PHS1, PRD2, PRD3 orthologs were mostly not detected in some distant algal lineages suggesting their minimal conservation. Remarkably, an ancestral duplication of SPO11 to all eukaryotes could be confirmed. Loss of SPO11-1 in Chlorophyta and Charophyta is likely to have occurred, suggesting that SPO11-1 and SPO11-2 heterodimerisation may be a unique feature in land plants of Viridiplantae. The possible origin of the meiotic proteins described only in plants till now, DFO and HEIP1, could be traced and seems to occur in the ancestor of vascular plants and Streptophyta, respectively. Our comprehensive approach is an attempt to provide insights about meiotic core proteins and thus the conservation of meiotic pathways across plant kingdom. We hope that this will serve the meiotic community a basis for further characterisation of interesting candidates in future.

**Supplementary Information:**

The online version contains supplementary material available at 10.1007/s00497-022-00456-1.

## Introduction

The mechanisms of meiosis, with a few notable exceptions, are highly conserved among sexually reproducing eukaryotes such as fungi, plants and animals (Gerton and Hawley 2005; Villeneuve and Hillers 2001). These processes include sister chromatid cohesion, homologous chromosome pairing, formation of the synaptonemal complex, double-stranded break (DSB) formation and processing, cross-over (CO) formation and resolution and two-step segregation of chromosomes, making meiosis special and different from mitosis. Therefore, typically, a common and shared set of specific meiotic genes can be found in all sexually reproducing organisms.

Formation of programmed double-stranded breaks (DSBs) during Prophase I is the upstream of many meiotic processes. First discovered in the budding yeast *Saccharomyces cerevisiae*, DSB initiation is catalysed by the highly conserved protein, SPO11 (Bergerat et al. 1997; de Massy et al. 1995; Keeney et al. 1997; Keeney and Kleckner 1995; Liu et al. 1995). In plants until now, many proteins have been isolated that function in DSB formation—PHS1/Rec114, PRD1/Mei1, PRD2/Mei4, PRD3/PAIR1/Mer2, DFO, PCH2 and MTOPVIB among which DFO have only been described in plants until now. DSBs are later loaded by the recombinases—RAD51 and DMC1. DMC1-mediated DNA repair using non-sister homologous chromatid appears to be the predominant pathway during *Arabidopsis thaliana* meiosis (Mercier et al. 2015). Chromosome axis mediates the formation of DSBs and its consecutive repair, resulting in the formation of inter-homolog COs. Cohesin complexes and axial element protein complexes form the components of chromosome axis formation. Cohesion complex is formed by the proteins—SMC1, SMC3, alpha-kleisin unit (SCC1/REC8) and SCC3 (Chelysheva et al. 2005; Onn et al. 2008). ASY1 and ASY2 are the HORMA domain containing axis proteins. ASY3 and ASY4 are the axis core proteins, essential for the recruitment of the HORMA domain proteins and the formation of axis (Caryl et al. 2000; Chambon et al. 2018; Ferdous et al. 2012; Sanchez-Moran et al. 2008, 2007; West et al. 2019). During the progression of prophase I, chromosome synapses and the axes of each homolog pair are connected to each other by coiled-coil transverse filaments (Dong and Roeder 2000; Liu et al. 1996; Meuwissen et al. 1992; Sym et al. 1993). ZYP1A and ZYP1B are identified as the proteins involved in the formation of synaptonemal complex (SC) in *A. thaliana* (Capilla-Perez et al. 2021; France et al. 2021; Higgins et al. 2005). There are two pathways for the formation of the COs—interference sensitive Class I and interference insensitive Class II pathways. Class I is the major one and depends on ZMM proteins (HEI10, HEIP1, MER3, MSH4, MSH5, PTD, ZIP2/SHOC1, ZIP4) and MLH1, MLH3 (Börner et al. 2004; Chelysheva et al. 2012; Dion et al. 2007; Franklin et al. 2006; Higgins et al. 2004, 2008b; Kuromori et al. 2008; Li et al. 2018; Lu et al. 2014; Macaisne et al. 2008; Mercier et al. 2005). Numerous DSBs are formed among which very few are processed to form COs. CO designation is still poorly understood (Berchowitz et al. 2007; Higgins et al. 2008a).

Understanding meiosis in plants can form a basis for advances in reproduction, fertility, genetics, breeding and thereby accelerate agricultural applications (Sanchez-Moran et al. 2008). Plants are also considered to be a good model system to study meiosis because in meiotic mutants, meiosis proceeds until the end of tetrad formation stage with meiotic defects like massive chromosome segregation defects but without confounding effects from the onset of meiotic arrest and apoptosis like in mammals (Higgins et al. 2004; Mercier and Grelon 2008). The kingdom Plantae or Archaeplastida in a broader sense includes freshwater unicellular algae (glaucophytes), photoautotrophic red algae (rhodophytes) and Viridiplantae which includes the paraphyletic group of green algae (chlorophytes and charophytes) and land plants. Land plants can be further classified into bryophytes (liverworts, hornworts, mosses), lycophytes, pteridophytes (ferns) and spermatophytes (gymnosperms and angiosperms) (Puttick et al. 2018). Plants are quite diverse and land plants alone are suggested to be approximately 500,000 species in comparison against 5400 mammalian species in total (Corlett 2016). Among plants, most studies investigating meiosis have been carried out in angiosperms, and the vast majority of studies characterising meiotic genes is done in the model plant *A. thaliana* and also in rice, maize, wheat, barley among others (Mercier and Grelon 2008). In total, around 100 genes involved in meiosis have been functionally studied in *A. thaliana* (Zhang et al. 2018). However, considering the diversity of plants, studying a few angiosperm models alone will not be sufficient to understand the evolution of meiosis in this kingdom. Functionally studying representative meiotic proteins from all plant lineages would be nearly impossible due to practical reasons. However, revolutionary advances in genomics means that sequence information is increasingly accumulating for many members of the Viridiplantae (green plants), and homology search can provide insights about the presence of meiotic machinery orthologs in a wide range of organisms.

To date, there is no comprehensive study that has aimed to search and detect core meiotic genes across all the main groups of the plant kingdom. Therefore, in this study, we searched for homologs of well-studied angiosperm meiotic genes among different plant lineages from algae to angiosperms. We bring to the attention of the readers that this paper discusses only Viridiplantae; however, rhodophytes and glaucophytes were included in our analysis as an outgroup. Our approach has allowed us to trace the conservation of the ancestral molecular machinery of plant meiosis and establish a correlation with the evolution of meiosis and the presence/absence of meiotic homologs across Viridiplantae. We found that proteins involved in DSB formation, chromosome axis formation and ZMM pathway are not detected in some early plant lineages, suggesting they are either missing or evolving rapidly during the diversification of the plant kingdom. Remarkably, our analysis confirms that land plants have two meiosis-expressed SPO11 paralogues, both essential for meiotic DSB formation and likely to act as a heterodimer, but only one homolog is retained in chlorophytes and charophytes. Our study shows how systematic analysis of the similarities and differences in meiotic regulation among plant species can provide insights into the fundamental elements of this critical process across evolution.

## Materials and methods

### Homology search using NCBI PSI-BLAST and phylogenetic tree construction

Twenty-seven genes with key meiotic function reported in *A. thaliana* were chosen for this study. Based on its function, the proteins were categorised into four pathways: chromosome axis/synaptonemal complex; double-strand break formation; strand invasion; and ZMM (Table 1). Protein sequences were downloaded from either UniProtKB or TAIR. TAIR has a list of plant homologs for all the proteins derived from the gene families of PANTHER 16.0 release which was used to create the initial multiple alignment file using MAFFT (Katoh and Standley 2013). NCBI PSI-BLAST was performed against selected species (Supplementary table 1) representing all plant lineages using *A. thaliana* protein sequence as the query. Initial MAFFT alignment was used as a PSSM upload. E-value threshold of maximum 5e-05 and BLOSUM62 matrix was the parameters used for the analysis. PSI-BLAST was continued by increasing the iteration until desired hits were obtained or until no significant hits were able to be found by PSI-BLAST. FASTA sequence of all the hits was downloaded, aligned by MAFFT, trimmed by trimAl (Capella-Gutierrez et al. 2009), and phylogenetic tree was constructed using IQ-TREE (Nguyen et al. 2015). In cases, where the tree could not be resolved, clustering analysis was performed using CLANS (Frickey and Lupas 2004). Cluster containing the initial query was filtered out, and the phylogenetic tree was constructed as described above. The trees were interpreted manually one by one.

**Table 1:** List of meiotic proteins used in this study

Protein id	Protein name in *Arabidopsis*	Alternative names in other species	Function in meiosis	Mutant phenotype	References	Sequence information
AT1G67370	ASY1	Hop1(yeast) HORMAD1 (mammals) PAIR2(rice)	Chromosome axis/SC	Failure in pairing, asynapsis or non-homologous synapsis, reduction in chiasma frequency	Caryl et al. 2000; Sanchez-Moran et al. 2008, 2007	> sp|F4HRV8|ASY1_ARATH Meiosis-specific protein ASY1 OS = Arabidopsis thaliana OX = 3702 GN = ASY1 PE = 1 SV = 1 MVMAQKLKEAEITEQDSLLLTRNLLRIAIFNISYIRGLFPEKYFNDKSVPALDMKIKKLM PMDAESRRLIDWMEKGVYDALQRKYLKTLMFSICETVDGPMIEEYSFSFSYSDSDSQDVM MNINRTGNKKNGGIFNSTADITPNQMRSSACKMVRTLVQLMRTLDKMPDERTIVMKLLYY DDVTPPDYEPPFFRGCTEDEAQYVWTKNPLRMEIGNVNSKHLVLTLKVKSVLDPCEDEND DMQDDGKSIGPDSVHDDQPSDSDSEISQTQENQFIVAPVEKQDDDDGEVDEDDNTQDPAE NEQQLARVKDWINSRHLDTLELTDILANFPDISIVLSEEIMDQLVTEGVLSKTGKDMYIK KRDKTPESEFTFVKEEADGQISPGKSVAPEDYLYMKALYHSLPMKYVTITKLHNMLDGEA NQTAVRKLMDRMTQEGYVEASSNRRLGKRVIHSSLTEKKLNEVRKVLATDDMDVDVTETI NKTNGPDAKVTADVSTCGGIHSIGSDFTRTKGRSGGMQQNGSVLSEQTISKAGNTPISNK AQPAASRESFAVHGGAVKEAETVNCSQASQDRRGRKTSMVREPILQYSKRQKSQAN
AT2G46980	ASY3	SYCP2(mammals) Red1(yeast) PAIR3(rice)	Chromosome axis/SC	Abnormal chromosome axis, disrupts SC formation, reduced meiotic COs, univalent formation and mis segregation of chromosomes	Ferdous et al. 2012	> sp|Q0WR66|ASY3_ARATH Meiosis-specific protein ASY3 OS = Arabidopsis thaliana OX = 3702 GN = ASY3 PE = 1 SV = 1 MSDYRSFGSNYHPSSQSRKISIGVMADSQPKRNLVPDKDDGDVIARVEKLKSATVTELQA NKKEKSDLAAKQRNSAQVTGHVTSPWRSPRSSHRKLGTLESVLCKQTSSLSGSKGLNKGL NGAHQTPARESFQNCPISSPQHSLGELNGGRNDRVMDRSPERMEEPPSAVLQQKVASQRE KMDKPGKETNGTTDVLRSKLWEILGKASPANNEDVNSETPEVEKTNFKLSQDKGSNDDPL IKPRHNSDSIETDSESPENATRRPVTRSLLQRRVGAKGVQKKTKAGANLGRKCTEQVNSV FSFEEGLRGKIGTAVNSSVMPKKQRGRRKNTVVKCRKAHSRKKDEADWSRKEASKSNTPP RSESTETGKRSSSSDKKGSSHDLHPQSKARKQKPDISTREGDFHPSPEAEAAALPEMSQG LSKNGDKHERPSNIFREKSVEPENEFQSPTFGYKAPISSPSPCCSPEASPLQPRNISPTL DETETPIFSFGTKKTSQGTTGQASDTEKRLPDFLEKKRDYSFRRESSPEPNEDLVLSDPS SDERDSDGSREDSPVLGHNISPEERETANWTNERSMLGPSSVKRNSNLKGIGRVVLSPPS PLSKGIDKTDSFQHCSEMDEDEDEGLGRAVALFAMALQNFERKLKSAAEKKSSEIIASVS EEIHLELENIKSHIITEAGKTSNLAKTKRKHAETRLQEQEEKMRMIHEKFKDDVSHHLED FKSTIEELEANQSELKGSIKKQRTSHQKLIAHFEGGIETKLDDATKRIDSVNKSARGKML QLKMIVAECLRDD
AT2G33793	ASY4	SYCP3(mammals)	Chromosome axis/SC	Defective chromosome axis, incomplete synapsis, reduction in formation of COs	Chambon et al. 2018	> tr|F4IFY5|F4IFY5_ARATH DNA ligase-like protein OS = Arabidopsis thaliana OX = 3702 GN = At2g33793 PE = 4 SV = 1 MSSTRRGTKRTRPEPPQSLKKPTPKAKLPDELDVDVSSDFKGIMSALQQFREKAHEDGRK KKEESISSVSTEVKSKIDELKSKLEKERQNFSKALSKSSKECENILKDEAAKFEELHKKF VKDKADHLQGLKDTISKFEEDKERLYMRYEQLRKKEKTMITEQEKFCTEKLAQLEESLKK KKRGDKTFSILRKTLGSFLENEASDEEFPPDE
AT5G05490	REC8/SYN1/DIF1	Rec8(yeast) REC8(mammals)	Chromosome axis/SC	Defects in SCC, defects in chromosome condensation leading to the formation of chromosome fragmentation, chromosome mis segregation, formation of univalent and aneuploids, sterility	Bai et al. 1999; Bhatt et al. 1999; Cai et al. 2003; Chelysheva et al. 2005; Peirson et al. 1997	> sp|Q9S7T7|SCC11_ARATH Sister chromatid cohesion 1 protein 1 OS = Arabidopsis thaliana OX = 3702 GN = SYN1 PE = 2 SV = 2 MLRLESLIVTVWGPATLLARKAPLGQIWMAATLHAKINRKKLDKLDIIQICEEILNPSVP MALRLSGILMGGVVIVYERKVKLLFDDVNRFLVEINGAWRTKSVPDPTLLPKGKTHARKE AVTLPENEEADFGDFEQTRNVPKFGNYMDFQQTFISMRLDESHVNNNPEPEDLGQQFHQA DAENITLFEYHGSFQTNNETYDRFERFDIEGDDETQMNSNPREGAEIPTTLIPSPPRHHD IPEGVNPTSPQRQEQQENRRDGFAEQMEEQNIPDKEEHDRPQPAKKRARKTATSAMDYEQ TIIAGHVYQSWLQDTSDILCRGEKRKVRGTIRPDMESFKRANMPPTQLFEKDSSYPPQLY QLWSKNTQVLQTSSSESRHPDLRAEQSPGFVQERMHNHHQTDHHERSDTSSQNLDSPAEI LRTVRTGKGASVESMMAGSRASPETINRQAADINVTPFYSGDDVRSMPSTPSARGAASIN NIEISSKSRMPNRKRPNSSPRRGLEPVAEERPWEHREYEFEFSMLPEKRFTADKEILFET ASTQTQKPVCNQSDEMITDSIKSHLKTHFETPGAPQVESLNKLAVGMDRNAAAKLFFQSC VLATRGVIKVNQAEPYGDILIARGPNM
AT1G22260 / AT1G22275	ZYP1 (*Arabidopsis* has ZYP1a and ZYP1b that are functionally redundant)	Zip1(yeast) SYCP1(mammals)	Chromosme axis/SC			
AT1G22260	ZYP1A			Reduction in fertility, Absence of SC, delayed prophase I, slight reduction in recombination, recombination between non-homologous chromosomes, absence of heterochiasmy	Capilla-Perez et al. 2021; France et al. 2021; Higgins et al. 2005	> sp|Q9LME2|SYCP1_ARATH Synaptonemal complex protein 1 OS = Arabidopsis thaliana OX = 3702 GN = ZYP1A PE = 2 SV = 1 MQKLGFPAMKSLDKPRSLSGSANMYSFSNRKPPDSVSSGSFSNLKLTAEKLVKDQAAMRT DLELANCKLKKSMEHVYALEEKLQNAFNENAKLRVRKKEDEKLWRGLESKFSSTKTLCDQ LTETLQHLASQVQDAEKDKGFFETKFSTSSEAIDSLNQQMRDMSLRLDAAKEEITSRDKE LEELKLEKQQKEMFYQTERCGTASLIEKKDAVITKLEASAAERKLNIENLNSQLEKVHLE LTTKEDEVKDLVSIQEKLEKEKTSVQLSADNCFEKLVSSEQEVKKLDELVQYLVAELTEL DKKNLTFKEKFDKLSGLYDTHIMLLQKDRDLALDRAQRSFDNLQGELFRVAATKEALESA GNELNEKIVELQNDKESLISQLSGLRCSTSQTIDKLESEAKGLVSKHADAESAISQLKEE METLLESVKTSEDKKQELSLKLSSLEMESKEKCEKLQADAQRQVEELETLQKESESHQLQ ADLLAKEVNQLQTVIEEKGHVILQCNENEKQLNQQIIKDKELLATAETKLAEAKKQYDLM LESKQLELSRHLKELSQRNDQAINEIRRKYDVEKHEIINSEKDKVEKIIKDLSNKFDKEL SDCKEESKRQLLTIQEEHSSLILSLREEHESKELNLKAKYDQELRQSQIQAENELKERIT ALKSEHDAQLKAFKCQYEDDCKKLQEELDLQRKKEERQRALVQLQWKVMSDNPPEEQEVN SNKNYSISKDSRLGGSKRSEHIRVRSDNDNVQDSPFVKAKETPVSKILKKAQNVNAGSVL SIPNPKHHSKVTHREYEVETNNGRVTKRRKTRNTTMFEEPQRRRTRATPKLTPQSIAKGT GMTSHARSANIGDLFSEGSLNPYADDPYAFD
AT1G22275	ZYP1B			Reduction in fertility, Absence of SC, delayed prophase I, slight reduction in recombination, recombination between non-homologous chromosomes, absence of heterochiasmy	Capilla-Perez et al. 2021; France et al. 2021; Higgins et al. 2005	> sp|P61430|SYCP2_ARATH Synaptonemal complex protein 2 OS = Arabidopsis thaliana OX = 3702 GN = ZYP1B PE = 2 SV = 1 MQKLGFPAMKSFDQLRSLPGSAKTYFFSTRPPQDSVSSGSFSNLKLTAEKLVKDQAAMRT DLELANCKLKKSMEHVYALEEKLQSAFNENAKLRVRQKEDEKLWRGLESKFSSTKTLCDQ LTETLQHLASQVQDAEKDKGFFETKFNTSSEAINSLNQQMRDMSLRLDAAKEEITSRDKE LEELKLEKQHKEMFYQTERCGTASLIEKKDAVITELETTAAERKLKIEKLNSQLEKLHLE LTTKEDEVIHLVSIQEKLEKEKTNVQLSSDELFEKLVRSEQEVKKLDELVHYLIAELTEL DKKNLTFKEKFDKLSGLYDTHFMLLRKDRDLASDRAQRSFDQLQGELFRVAAEKEALESS GNELSEKIVELQNDKESLISQLSGVRCSASQTIDKLEFEAKGLVLKNAETESVISKLKEE IDTLLESVRTSEDKKKELSIKLSSLEIESKDKYEKLQADAQRQVGELETLQKESESHQLQ ADLLAKEVNQLQTIIEEKGHLILQCNENEKNINQQIIKDKELLATAETKLAEAKKQYDLM LESKQLELSRHLKELSQRNDQAINEIRRKYDVEKHEIINSEKDKVEKIIKELSTKYDKGL SDCKEESKRQLLTIQEEHSSRILNIREEHESKELNLKAKYDQELRQNQIQAENELKERIT ALKSEHDAQLKAFKCQYEDDCKKLQEELDLQRKKEERQRALVQLQWKVMSDNPPEEQEVN SNKDYSHSSVKVKESRLGGNKRSEHITESPFVKAKVTSVSNILKEATNPKHHSKVTHREY EVETNNGRIPKRRKTRQTTMFQEPQRRSTRLTPKLMTPTIIAKETAMADHPHSANIGDLF SEGSLNPYADDPYAFD
AT1G07060	DFO	Reported to be plant specific so far	DSB	Reduced fertility, formation of polyads, defects in chromosome synapsis and segregation and recombination	Zhang et al. 2012	> sp|Q8RX33|DFO_ARATH Protein DOUBLE-STRAND BREAK FORMATION OS = Arabidopsis thaliana OX = 3702 GN = DFO PE = 1 SV = 1 MRHNIKFKSKGTLKIRNTAQISLWKKCSDSMIADQTYLFINRVQDRRFDEESLRILELSL VAMNVKSFLEVRSRLRDFMRSESVVIFGELTGESMVAKLSVLEFFARAFALLGDMESCLA MRYEALNLRQLKSPSCLWLGVSHSEWTKFAVQSMENGFPSIAGKASENALLSLKKDSLIE PKSEDNSDILDAAEKVRRLRDSAASLTSSHSGIFIYIVSSLKFAVCNRLLTTF
AT1G60460	MTOPVIB	TOPOVIBL (mouse)	DSB	Reduction in fertility, defects in synapsis and recombination	Tang et al. 2017; Vrielynck et al. 2016	> sp|Q5Q0E6|TO6BL_ARATH Type 2 DNA topoisomerase 6 subunit B-like OS = Arabidopsis thaliana OX = 3702 GN = MTOPVIB PE = 1 SV = 1 MENNAPVPKLLLQLISSAFQRCRLAEDLCRLSVLLDQSTERDPPITCISIADTGIGCNLE EFQNLRCPREFNGAKIWDGLLSVKTTCFTDDEVYYYHINLDEYIANKRLKRQPSQAKNGA KFSGTEVSLSVFGSMDVLVAPIIGFFQKIIVLQILNVTLDLMVKQGTSPGNQTQYVFAVN ADKTPCFTASNLERLKSGLEDYVLRHANCLDTMCDYCFSDREHLKVGSGTVCQEDKHKRV GGTMEVVIVISDLLESTQHCSRSCNGKTEVLYFDNFLPSPVPHLALSALKKIDWKKYGLI LANVNDQDGHVFLEWDNFPSYVQIQIALHWYHNQYPTRQKNGPGISLLKKGIKNALDNLK AKHEGFLLSSHSRKICSYVPDLARSIAGLIFSSTDLDFQGDCLSVLGFQTQEVERDTVEN YIQRKIVTVIGMNERKPQKDQEAAPFLFFDGESETSFFEDEEVEGEYYSSSLE
AT1G10710	PHS1	REC114(mammals) Rec114 (Saccharomyces cerevisiae) Rec7 (Schizosaccharomyces pombe)	DSB	Defects in pairing—pairing of non-homologous chromosomes	Ronceret et al. 2009	> sp|Q45GQ7|POHS1_ARATH Protein POOR HOMOLOGOUS SYNAPSIS 1 OS = Arabidopsis thaliana OX = 3702 GN = PHS1 PE = 1 SV = 1 MAGSLTASNRRRNAEDSSEIYRWTIGFARFVHYPSSPSPHPVLKPLGKREQYHSPHGTWL SASSSTVSLHIVDELNRSDVILSVKLGQKVLEEHYISKLNFTWPQMSCVSGFPSRGSRAI FVTYMDSANQIQKFALRFSTCDAALEFVEALKEKIKGLKEASTQNQKNKTRCDVSFQSDY NPSDAIIPRATQKEPNMVRPLNSYVPEMLPRIVYEAQYQKSETRSEVSFQSDYNPSIEIF PRATEEEPNMVRFFDSSVPEVLPRPEYEAGQALYPSQSTLNQIPSLPPSFTTLLSGCFPD STLDAGQTTVKQNPDLKSQILKYMEDSSFQDMLQKVERIIDEIGGNWIT
AT4G14180	PRD1	MEI1(mammals)	DSB	Defects in synapsis, reduction of recombination rates, formation of achiasmatic univalents	De Muyt et al. 2007	> sp|O23277|PRD1_ARATH Protein PUTATIVE RECOMBINATION INITIATION DEFECT 1 OS = Arabidopsis thaliana OX = 3702 GN = PRD1 PE = 1 SV = 3 MFFQHSQLQNSDHLLHESMADSNHQSLSPPCANGHRSTISLRDDQGGTFCLICFSNLVSD PRIPTVHVSYALHQLSIAISEPIFLRTLLSSHIHFLVSPLVHALSSIDDAPIAIQIMDMI SLLCSVEESSIGEDFVERISDQLSSGALGWSRRQLHMLHCFGVLMSCENININSHIRDKE ALVCQLVEGLQLPSEEIRGEILFALYKFSALQFTEQNVDGIEVLSLLCPKLLCLSLEALA KTQRDDVRLNCVALLTILAQQGLLANSHSNSASSMSLDEVDDDPMQTAENVAARPCLNVL FAEAIKGPLLSTDSEVQIKTLDLIFHYISQESTPSKQIQVMVEENVADYIFEILRLSECK DQVVNSCLRVLDLFSLAEHSFRKRLVIGFPSVIRVLHYVGEVPCHPFQIQTLKLISSCIS DFPGIASSSQVQEIALVLKKMLERYYSQEMGLFPDAFAIICSVFVSLMKTPSFGETADVL TSLQESLRHSILASLSLPEKDSTQILHAVYLLNEVYVYCTASTSINKTICIELRHCVIDV CTSHLLPWFLSDVNEVNEEATLGIMETFHSILLQNSDIQAKEFAELLVSADWFSFSFGCL GNFCTDNMKQRIYLMLSSLVDILLEQKTGSHIRDALHCLPSDPQDLLFLLGQASSNNQEL ASCQSAALLIFHTSSIYNDRLADDKLVLASLEQYIILNKTSLICAISDSPALLNLVNLYG LCRSLQNERYQISYSLEAERIIFHLLNEYEWDLGSINIHLESLKWLFQQESISKSLIYQI QKISRNNLIGNEVHNVYGDGRQRSLTYWFAKLISEGDNYAATLLVNLLTQLAEKEEQEND VISILNLMNTIVSIFPTASNNLSMNGIGSVIHRLVSGFSNSSLGTSFRTLLLLVFNILTS VQPAVLMIDESWYAVSIKLLNFLSLRDTAIKQNHEDMVVIGILSLVLYHSSDGALVEASR NIVSNSYLVSAINTVVDVACSKGPALTQCQDETNIGEALAFTLLLYFFSLRSLQIVLAGA VDWQTFFGTSTSLETLPVVCIHCHNLCRLMHFGAPQIKLIASYCLLELLTGLSEQVDIKK EQLQCSSSYLKSMKAVLGGLVFCDDIRVATNSALCLSMILGWEDMEGRTEMLKTSSWYRF IAEEMSVSLAMPCSASSTYVNHHKPAVYLTVAMLRLKNKPVWLRTVFDESCISSMIQNLN GINISREIVILFRELMQAELLNSQQVTKLDRAFQECRKQMHRNGTRDETVEEQVQRKIPS IHDHSEFCNYLVHLMVSNSFGHPSESETYTQKKKQILDEMEQFSELISTREGRVSPIQEE TRQMQTERIV
AT5G57880	PRD2/MPS1	MEI4(mammals) Mei4(S. cerevisiae) Rec24(S. pombe)	DSB	Aberrant spindle formation and disordered chromosome segregation	Jiang et al. 2009	> sp|F4KDF5|MUPS1_ARATH Protein MULTIPOLAR SPINDLE 1 OS = Arabidopsis thaliana OX = 3702 GN = MPS1 PE = 2 SV = 2 MSSSVAEANHTEKEESLRLAIAVSLLRSKFQNHQSSSSTSRCYVSSESDALRWKQKAKER KKEIIRLQEDLKDAESSFHRDLFPANASCKCYFFDNLGVFSGRRIGEASESRFNDVLRRR FLRLARRRSRRKLTRSSQRLQPSEPDYEEEAEHLRISIDFLLELSEADSNDSNFSNWSHQ AVDFIFASLKKLISMGRNLESVEESISFMITQLITRMCTPVKGNEVKQLETSVGFYVQHL IRKLGSEPFIGQRAIFAISQRISILAENLLFMDPFDESFPEMDECMFILIQLIEFLICDY LLPWANEAFDNVMFEEWIASVVHARKAVKALEERNGLYLLYMDRVTGELAKRVGQITSFR EVEPAILDKILAYQEIE
AT1G01690	PRD3	Mer2(S. cerevisiae) Rec15(S. pombe)	DSB	Defects in DSB formation leading to defects in synapsis and chiasma formation	De Muyt et al. 2009	> sp|Q0WWX5|PRD3_ARATH Putative recombination initiation defects 3 OS = Arabidopsis thaliana OX = 3702 GN = PRD3 PE = 1 SV = 2 MKMNINKACDLKSISVFPPNLRRSAEPQASQQLRSQQSQQSFSQGPSSSQRGCGGFSQMT QSSIDELLINDQRFSSQERDLSLKKVSSCLPPINHKREDSQLVASRSSSGLSRRWSSASI GESKSQISEELEQRFGMMETSLSRFGMMLDSIQSDIMQANRGTKEVFLETERIQQKLTLQ DTSLQQLRKEQADSKASLDGGVKFILEEFSKDPNQEKLQKILQMLTTIPEQVETALQKIQ REICHTFTREIQVLASLRTPEPRVRVPTAPQVKAKENLPEQRGQAAKVLTSLKMPEPRVQ VPAAPQAKENFPEQRGPVAKSNSFCNTTLKTKQPQFPRNPNDASARAVKPYLSPKIQVGC WKTVKPEKSNFKKRATRKPVKSESTRTQFEQCSVVIDSDEEDIDGGFSCLINENTRGTNF EWDAEKETERILRTARRTKRKFGNPIIIN
AT3G13170	SPO11-1	SPO11(mammals) Spo11(S. cerevisiae) Rec12(S. pombe)	DSB	Defects in synapsis, bivalent formation, meiotic recombination reduction, defects in chromosome segregation, semi-sterile phenotype	Grelon et al. 2001	> sp|Q9M4A2|SPO11_ARATH Meiotic recombination protein SPO11-1 OS = Arabidopsis thaliana OX = 3702 GN = SPO11-1 PE = 1 SV = 1 MEGKFAISESTNLLQRIKDFTQSVVVDLAEGRSPKISINQFRNYCMNPEADCLCSSDKPK GQEIFTLKKEPQTYRIDMLLRVLLIVQQLLQENRHASKRDIYYMHPSAFKAQSIVDRAIG DICILFQCSRYNLNVVSVGNGLVMGWLKFREAGRKFDCLNSLNTAYPVPVLVEEVEDIVS LAEYILVVEKETVFQRLANDMFCKTNRCIVITGRGYPDVSTRRFLRLLMEKLHLPVHCLV DCDPYGFEILATYRFGSMQMAYDIESLRAPDMKWLGAFPSDSEVYSVPKQCLLPLTEEDK KRTEAMLLRCYLKREMPQWRLELETMLKRGVKFEIEALSVHSLSFLSEVYIPSKIRREVS SP
AT1G63990	SPO11-2	SPO11(mammals) Spo11(S. cerevisiae) Rec12(S. pombe)	DSB	Severe defects in synapsis, reduction in meiotic recombination, sterility	Stacey et al. 2006	> sp|Q9M4A1|SPO12_ARATH Meiotic recombination protein SPO11-2 OS = Arabidopsis thaliana OX = 3702 GN = SPO11-2 PE = 1 SV = 1 MEESSGLSSMKFFSDQHLSYADILLPHEARARIEVSVLNLLRILNSPDPAISDLSLINRK RSNSCINKGILTDVSYIFLSTSFTKSSLTNAKTAKAFVRVWKVMEICFQILLQEKRVTQR ELFYKLLCDSPDYFSSQIEVNRSVQDVVALLRCSRYSLGIMASSRGLVAGRLFLQEPGKE AVDCSACGSSGFAITGDLNLLDNTIMRTDARYIIIVEKHAIFHRLVEDRVFNHIPCVFIT AKGYPDIATRFFLHRMSTTFPDLPILVLVDWNPAGLAILCTFKFGSIGMGLEAYRYACNV KWIGLRGDDLNLIPEESLVPLKPKDSQIAKSLLSSKILQENYIEELSLMVQTGKRAEIEA LYCHGYNYLGKYIATKIVQGKYI
AT1G13330	HOP2/AHP2	Hop2(S. cerevisiae) Meu13(S. pombe)	Strand invasion	Sterility, defects in bivalent formation, chromosome fragmentation, chromatin bridges, unbalanced segregation	Schommer et al. 2003	> sp|Q9FX64|HOP2_ARATH Homologous-pairing protein 2 homolog OS = Arabidopsis thaliana OX = 3702 GN = HOP2 PE = 1 SV = 1 MAPKSDNTEAIVLNFVNEQNKPLNTQNAADALQKFNLKKTAVQKALDSLADAGKITFKEY GKQKIYIARQDQFEIPNSEELAQMKEDNAKLQEQLQEKKKTISDVESEIKSLQSNLTLEE IQEKDAKLRKEVKEMEEKLVKLREGITLVRPEDKKAVEDMYADKINQWRKRKRMFRDIWD TVTENSPKDVKELKEELGIEYDEDVGLSFQAYADLIQHGKKRPRGQ
AT4G29170	MND1	Mnd1(yeast) MND1(mammals)	Strand invasion	Failure in pairing and synapsis, chromosome fragmentation, mis segregation, formation of inviable gametes, sterility	Kerzendorfer et al. 2006; Panoli et al. 2006	> sp|Q8GYD2|MND1_ARATH Meiotic nuclear division protein 1 homolog OS = Arabidopsis thaliana OX = 3702 GN = MND1 PE = 1 SV = 1 MSKKRGLSLEEKREKMLQIFYESQDFFLLKELEKMGPKKGVISQSVKDVIQSLVDDDLVA KDKIGISIYFWSLPSCAGNQLRSVRQKLESDLQGSNKRLAELVDQCEALKKGREESEERT EALTQLKDIEKKHKDLKNEMVQFADNDPATLEAKRNAIEVAHQSANRWTDNIFTLRQWCS NNFPQAKEQLEHLYTEAGITEDFDYIELSSFPLSSSHEADTAKQLVQDEA
AT3G22880	DMC1	Dmc1(yeast) DMC1(mammals)	Strand invasion	Formation of univalents and reduced fertility	Couteau et al. 1999; Crismani et al. 2013	> sp|Q39009|DMC1_ARATH Meiotic recombination protein DMC1 homolog OS = Arabidopsis thaliana OX = 3702 GN = DMC1 PE = 1 SV = 2 MMASLKAEETSQMQLVEREENDEDEDLFEMIDKLIAQGINAGDVKKLQEAGIHTCNGLMM HTKKNLTGIKGLSEAKVDKICEAAEKIVNFGYMTGSDALIKRKSVVKITTGCQALDDLLG GGIETSAITEAFGEFRSGKTQLAHTLCVTTQLPTNMKGGNGKVAYIDTEGTFRPDRIVPI AERFGMDPGAVLDNIIYARAYTYEHQYNLLLGLAAKMSEEPFRILIVDSIIALFRVDFTG RGELADRQQKLAQMLSRLIKIAEEFNVAVYMTNQVIADPGGGMFISDPKKPAGGHVLAHA ATIRLLFRKGKGDTRVCKVYDAPNLAEAEASFQITQGGIADAKD
AT4G24710	PCH2	Pch2(yeast) PCH2/TRIP13 (mammals) CRC1(rice)	Strand invasion	Defects in CO maturation leading to the formation of univalents	Lambing et al. 2015	> sp|Q8H1F9|PCH2_ARATH Pachytene checkpoint protein 2 homolog OS = Arabidopsis thaliana OX = 3702 GN = PCH2 PE = 2 SV = 1 MVEDPIPLPNASMEVSYQNPIEAATIPVQIAVAEPVATPNPPPCLHENKFLVSVEVCLKP SSTARLEDVQRAVERMLENRSMSYADGLVLIPADDLFLVDNVQRICICDTEEWVKNNDVL LFWQVKPVVHTFQLIEEGPCEDLCADGQPASFNEWILPAKEFDGLWESLIYESGLKQRLL RYAASALLFTQKGVNPNLVSWNRIILLHGPPGTGKTSLCKALAQKLSIRCNSRYPHCQLI EVNAHSLFSKWFSESGKLVAKLFQKIQEMVEEDGNLVFVLIDEVESLAAARKAALSGSEP SDSIRVVNALLTQMDKLKSAPNVIILTTSNITTAIDVAFVDRADIKAYVGPPTLHVRYEI LRSCVEELISKGIISSFQGCDGLSIPSFSSLKEKLSESEVHDTNTVPWFCKQLIEAAKGC EGLSGRSLRKLPFLAHAALADPYSHDPSNFLCTMIETAKREKSEQPE
AT1G53490	HEI10	HEI10/RNF212 (mammals) Zip3/Hei10(yeast)	ZMM	Asymmetric tetrads or polyads, fertility defects leading to reduced seed number per silique	Chelysheva et al. 2012	> sp|F4HRI2|HEI10_ARATH E3 ubiquitin-protein ligase CCNB1IP1 homolog OS = Arabidopsis thaliana OX = 3702 GN = HEI10 PE = 2 SV = 1 MRCNACWRDLEGRAISTTCGHLLCTEDASKILSNDGACPICDQVLSKSLMKPVDINPNEE WINMAMAGISPQILMKSAYRSVMFYIAQRDLEMQYKMNRVVAQCRQKCEGMQAKFSEKME QVHTAYQKMGKRCQMMEQEVENLTKDKQELQEKFSEKSRQKRKLDEMYDQLRSEYESVKR TAIQPANNFYPRHQEPDFFSNPAVNMMENRETIRKDRSFFSPATPGPKDEIWPARQNSSN SGPFDISTDSPAIPSDLGNRRAGRGHPVYGGGGTANPQSTLRNLILSPIKRSQLSRSRPQ LFTL
AT2G30480	HEIP1	Reported to be plant specific so far	ZMM	Severe reduction of chaisma frequency, formation of univalent, sterility	Li et al. 2018	> tr|F4INT5|F4INT5_ARATH Uncharacterised protein OS = Arabidopsis thaliana OX = 3702 GN = At2g30480 PE = 4 SV = 1 MLQWMGGSRRKVAASHTSVKKRQKQYFEQRRQQQHQFTVGSESCSNDINNSNQHLREHQS LDILNLLNLSTATPECKPSGPENGMQDLDADFYSLKDNMSGVGSSFNHIAEPTSSKRTLF SIPDNQTNDFKKANTDNQTNDFKKTNTTADLMDGTERKLSVFDLVGDDHTTTNLEECSPS EAHMAFSVEGLGKINTETPVNSPQPSDRTFVYRCSSPWKDTGQPDTSHVRGRLNDFENEV DTMIQSSKMFQDDSLYRSPIGIHAKDGGRKQKLQTFSDHLHKQYSDSRNYFCDVADFNNS RFSDDEWNAKPAFLDDGEDSFYWKAEQPCQKESLNPDFLKYCNDCTESRSSTEHHRKKKR DYLETTWRSNIRDSPTRRSHLLKRNIDYPSFAKAATSDFDFDNVFDRPVWSSIVLEEDKD SHSLRSEESCSSSAVWTNETHNSQFETNTRQRKRETNKFSNLGDKKYINTDLFQESWEDW EVDDQHMKRQVRSGKQGRLSNSGKLKSTSQRKGGLDASYDWFGEGFTSAGINSEITSERN KPYPFLNPERGSSHWRSSRAPDSIPETWIPKFSVGGTGDDDDGDHDEEDYVNCLSANHKS KLAGDTCGFENDTLSENDNEQSREVNHPKNQGDETSSSIAKSLSDENDVVRCNPNKEVME ARHQRNRESGEKTSRDPFQQMIMLERRTLQLVCFNKALLLDSLKT
AT3G27730	MER3, RCK, ROCK-N-ROLLERS	Mer3(yeast) HFM1(mammals)	ZMM	Low levels of fertility due to defects in synapsis and CO formation	Chen et al. 2005; Mercier et al. 2005	> sp|Q5D892|MER3_ARATH DExH-box ATP-dependent RNA helicase DExH17 OS = Arabidopsis thaliana OX = 3702 GN = MER3 PE = 2 SV = 1 MDTHTLKSVSDLPGNFRSAFSFRYFNSLQSECFPLCFHSDINMIISAPTGSGKTVLFELC ILRLFSKSISKEGSFLHAKGALKTVYISPSKALVQEKLRDWNQKFNSWGISCLELTGDNE TYSTKNIQDADIILTTPEKFDAVSRYRVTSGGLGFFSDIALVLIDEVHLLNDPRGAALEA IVSRLKILSSNHELRSSTLASVRLLAVSATIPNIEDLAEWLKVPTAGIKRFGEEMRPVKL TTKVFGYAAAKNDFLFEKRLQNYIYDILMQYSKGKSALVFCSTRKGAQEAAQKLAQTAMT YGYSNPFIKSREQLERLREASPMCSDKQMQSYILQGVGYHNGGLCQKDRSLVEGLFLNGD IQVICTTNTLAHGINLPAHTVVIKSTQHFNKEKGHYMEYDRSTLLQMSGRAGRPPFDDTG MVIIMTRRETVHLYENLLNGCEVVESQLLPCLIEHLTAEIVQLTISDITRAIEWMKCSYL YVRMKKNPENYAIKKGIPKDRVEKHLQELCLQKINELSQYQMIWTDTDGFVLKPEEPGRL MTKYYLKFETMKYIINTPTSYSLDEALHIVCHAEEISWIQLRRNEKKTLNDVNADKEGRL RFHINDNKGKRKKRIQTREEKLFVLANDWLTGDPSVHDLSMTQDANSICSNGSRIARCMK EYFIYKKNYKGTLSSTLLAKSLYQKLWDDSPYLLKQLPGIGMVTAKALHSMGVRSFEALA EADPRRIEIVTGRKYPFGNTIKESLSSLPPKVEIKVEEVDCQKQGISKLAVTLSRVSQPL QSTKRHYADLIVGSEEENLIHFHEKIRMEDFSSPYSVTVLLERPHQQTKVTVKADLIFEE YIGIDLHETLLLKKANNNKVNYKSENRMPQYYPPMASACIADDDNPVTSGPSNRKDKKDD MPSFKLIDDDSEEEKEPYVTMEEDDCVIINEHTVFDHIREKAKCFPSLNPLNPTSSPASG KSILKRKSLVENNSPELDPLFQYDSVFDLPTNTKDIKQSAQQITSPGYASFAEKTETERP FSDETIFNYIRKRSKNSPALATSKIENPITISSQEGRNAEISPYRTYGLLVSPATKIPRI TSDAPSEILSFDISMVKRSDTSLEQTKGFCSTLAGKSNVSDSFLGFKSIFSFL
AT4G17380	MSH4	Msh4(yeast) MSH4(mammals)	ZMM	Delayed/incomplete synapsis but doesn't prevent meiosis, reduction in chiasma frequency, formation of univalents, nondisjunction of chromosomes leading to severe reduction in fertility	Higgins et al. 2004	> sp|F4JP48|MSH4_ARATH DNA mismatch repair protein MSH4 OS = Arabidopsis thaliana OX = 3702 GN = MSH4 PE = 2 SV = 1 MEDDGGERSSFVAGLIENRAKEVGMAAFDLRSASLHLSQYIETSSSYQNTKTLLRFYDPS VIIVPPNKLAADGMVGVSELVDRCYSTVRKVVFARGCFDDTKGAVLIQNLAAEEPLALGL DTYYKQHYLSLAAAAATIKWIEAEKGVIVTNHSLTVTFNGSFDHMNIDATSVENLELIDP FHNALLGTSNKKRSLFQMFKTTKTAGGTRLLRANLLQPLKDIETINTRLDCLDELMSNEQ LFFGLSQVLRKFPKETDRVLCHFCFKPKKVTEAVIGFENTRKSQNMISSIILLKTALDAL PILAKVLKDAKCFLLANVYKSVCENDRYASIRKKIGEVIDDDVLHARVPFVARTQQCFAL KAGIDGFLDIARRTFCDTSEAIHNLASKYREEFNLPNLKLPFNNRQGFFFRIPQKEVQGK LPNKFTQVVKHGKNIHCSSLELASLNVRNKSAAGECFIRTETCLEALMDAIREDISALTL LAEVLCLLDMIVNSFAHTISTKPVDRYSRPELTDSGPLAIDAGRHPILESIHNDFVSNSI FMSEATNMLVVMGPNMSGKSTYLQQVCLVVILAQIGCYVPARFATIRVVDRIFTRMGTMD NLESNSSTFMTEMRETAFIMQNVTNRSLIVMDELGRATSSSDGLAMAWSCCEYLLSLKAY TVFATHMDSLAELATIYPNVKVLHFYVDIRDNRLDFKFQLRDGTLHVPHYGLLLAEVAGL PSTVIDTARIITKRITDKENKRIELNCGKHHEIHRIYRVAQRLICLKYSRQTEDSIRQAL QNLNESFTEERL
AT3G20475	MSH5	Msh5(yeast) MAH5(mammals)	ZMM	Reduction in chiasma frequency, formation of univalents, reduction in fertility	Higgins et al. 2008a, b; Lu et al. 2008	> sp|F4JEP5|MSH5_ARATH DNA mismatch repair protein MSH5 OS = Arabidopsis thaliana OX = 3702 GN = MSH5 PE = 2 SV = 1 MEEMEDTETEPQVYMACIQHGRRVGVSYYDCSVRQLHVLEFWEEDCSDFTLINMVKYQAK PSIIYASTKSEESFVAALQQNDGTDETTMVKLVKSSTFSYEQAWHRLVYLRVTGMDDGLN IKERICYLSSMMDVGSEVQVRVSGGLLAILESERIVETLEQNESGSASIAIDSVMEVPLN KFLKLDAAAHEALQIFQTDKHPSHMGIGRAKEGFSVFGMMNKCATPMGRRLLRSWFMRPI LDLEVLDRRLNAISFFISSVELMASLRETLKSVKDISHLLKKFNSPTSLCTSNDWTAFLK SISALLHVNKIFEVGVSESLREHMRRFNLDIIEKAGLCISTELDYVYELVIGVIDVTRSK ERGYQTLVKEGFCAELDELRQIYEELPEFLQEVSAMELEHFPHLHKEKLPPCIVYIQQIG YLMCIFGEKLDETALNRLTEFEFAFSDMDGETQRFFYHTSKTRELDNLLGDIYHKILDME RAIIRDLLSHTLLFSAHLLKAVNFVAELDCILSLACVAHQNNYVRPVLTVESLLDIRNGR HVLQEMAVDTFIPNDTEINDNGRIHIITGPNYSGKSIYVKQVALIVFLSHIGSFVPADAA TVGLTDRIFCAMGSKFMTAEQSTFMIDLHQVGMMLRQATSRSLCLLDEFGKGTLTEDGIG LLGGTISHFATCAEPPRVVVCTHLTELLNESCLPVSEKIKFYTMSVLRPDTESANMEEIV FLYRLIPGQTLLSYGLHCALLAGVPEEVVKRAAIVLDAFESNNNVDKLSLDKISSQDQAF KDAVDKFAELDISKGDIHAFFQDIFTS
AT1G12790	PTD	Spo16(yeast) SPO16(mammals)	ZMM	Reduction in the number of chiasmatas	Lu et al. 2014; Macaisne et al. 2011; Wijeratne et al. 2006	> sp|F4IDW9|PTD_ARATH Protein PARTING DANCERS OS = Arabidopsis thaliana OX = 3702 GN = PTD PE = 1 SV = 1 MATAGSSYSVSTDHQVSSPLVNLGNVAGVCIMSNAWKVEQEPSLINFISAFLSANSFRLN FVSIPPDLIFNCGGVSIAFVFVTKWDFSNVASIFSRVKRLKGQFAQLYVVATLSTKEQSD SFMRSYFQYEMEFGKPAFVQVTDAEMGFEKIVKIAHSRGVCKQQKVASKLKVERKRTVQD TNIFIRFVTSIPNINKHDANTLYQAIGSIEAIAKASKEDILANTDLSSKKADTLTRFFQD PEFYLSPKFN
AT5G52290	SHOC1, ZYP2	Zip2(yeast) SHOC1/MZIP2 (mammals)	ZMM	Reduction in the number of COs	Macaisne et al. 2008	> sp|F4KG50|SHOC1_ARATH Protein SHORTAGE IN CHIASMATA 1 OS = Arabidopsis thaliana OX = 3702 GN = SHOC1 PE = 1 SV = 1 MRTRFLNIDYFSTPPSHVFETLGFLNLPAPDNFPAPIVYNGEEDRLRFGSIENVSIPIGN LPIEAALSKFLSDVVPDRVSVDYRVFEIDDSSLGVYYSDEKDDGDAIADKATPKIIELET PELDFEMENKLLCTSEDHLQCFSEVLEIKNDPVKYEGSDIILQNSKDIQEQIYSVDYIPS DYFTENNTSVAENECFRKIQPWFKDARFPLLEVDEVNLSELSSLSVLDKVFTVLETIEPQ DTNAGSSLIINSKELIGSKDYDLLDVLSTDCYLNKSGQSDVVPEDEFSEMDIVTILEISN AEEFQGKVAVPVTYEEFQILDVDISDVFDIFLCLQKAIEPEICYGMFSKEMNFKDFDELV VSSELAFTDDAFKSLPTPILHDYEMTRSLELIYEDVLSKIKPQSLSASNDIYLPWNLLEE RNHNHCDYPFEEIVTFNIDYNWEASEGDKWVYDFIFSEDAFCEPLVEKCTEPFYGISNLD EHAPVNTSHGLLENPFQKTGARDCAVDDNAKKATLLFKSMSAFDDLTFFMDPKKAVIEDN LESRVEAAKTTNHKCMSIDSKASCRSGGMHPNPKTEEMILHSVRPSENIQALVGEFVKSY LTLVKDESENLSEDKLKLLSISKGKLIDCIRKANVHKTQLADDKTFTFALLLAIKQMTWY MCFFGIHVAYIYLNKVCRSSNPMKIGLHTLYSAVETEHKSDETDITRSHPSLAVIQGILQ SEFARGNSKALLLAEKVFWSSLKRLLMSMGLSYNDLNSPSPSGNRPNVHEAIELGFLPIS DCLIISYEQISPSFPVENFSVIVEYGGPNASPRYSFPSKLDSFPSFHFIKVELDMPSACG QLCAGVTVPYSLKMIKGDEVETKTGWLEEVLNFVPLEKVCYAGSSETTNESEFISMPQES ERKRGIIEQGLSDQRSVIVVNTKTVDKEMIISRRSTYQKVLAMEKEGVQVVERDSDLPVD LMLSPAVCLLWYDSETVSKKSAATIGTSSSSLSWIGDIATNVLTSLSFSFSTCIMVFEGE PAFLAAVMDSSDELYAAAGSLGISLQMFCSSSANLTDEIILKCIKSSVKLSKLHVKMPES ESLAESFLTKFPSVNPLTAQVILSSSGSLLEFMKLPHKSKVERTQKYHVPEESVDLFSSV CRYGAREDSRSVMTDSSSSVSSGPDSDTHHVSVHSGSKKKQYIAEKDEIDMDDLVHFSPS IEFADTQLKSSGDFQLDDSWSSKDHEIFHFDPVTEFSDAPFKPSGISHPNDSWPSKDPER FDKKSGPGSSSKDTFWEKDQPDFSVEDSLPGIPELEDWSFPVKDKFMSQNRGCKFPVMRD FNLHDNRNSENFIADYKGEVIDRADKYLEEDFPPSPGYNRFARIVSDVNEEELPRKSKSS RKLSFFGSLQPNFPKAADIDSSSERYATEKDSKYDNNTSLRGYADNYPAKRQRTLLEEVL TRRSAVPTTELPFREEISHFGGSPLSNAIRSSNQVQSSPWTVDFLNRVRERSRARKQQQS LPSYASPPSLETPGNIKKANTKRKSPSILEFFKYKGGNKLQEEKRQKRSKNSSASPKNER FYSPLKSCTPIDKRAKQSLSYTANGTGQTKLVWK
AT5G48390	ZIP4	Zip4/Spo22(yeast) TEX11(mouse)	ZMM	Reduction in the formation of COs	Chelysheva et al. 2007; Kuromori et al. 2008	> sp|B0M1H3|ZIP4L_ARATH TPR repeat-containing protein ZIP4 OS = Arabidopsis thaliana OX = 3702 GN = ZIP4 PE = 2 SV = 1 MRIAEITTPDLRLHHRETDSHTHHPLLSEIELLIQQSEAISKDQPLPQSLPISLRQFLTR LSQLAPFPDNSFKLTIWKLSFRLWNACVDLANAASLQSSLTSAENIANLRHVAADMLFLA KDVTGVPSPTIKSSLFYYKTGLVYHSLKKFDLASDCFERATEIVSKIDIAKISDAGEKKL FLDLNLARSRTAWEISDRNLAVTLLNRAKNLLFGSPDHYKSLSNQFLAFGKSSLSRGDDD CSLNDALRLMNEALDLCEKGLGTAKTREDTTEFTAMRIKTLRFISAVHLQKGEFENVIKC VKVLRNGGNGSDGADQHASLPVLAMKAWLGLGRHSEAEKELRGMVGNNDIPEAVWVSAVE AYFEVVGTAGAETAKGVFLGLLGRCHVSAKAALRVAHRVLGESRGGDNGSRIRANVVAQL VSDERVVALFASEAVTKERKAIHSVLWNSASDHFRAKDYETSAEMFEKSMLYIPHDIENR VFRAKGFRVLCLCYLGLSQLDRALEYIEEAEKLEPNIACSFLKFKIYLQKKEHSCAIGQI DAMTSCLDFSPDYLSLSAHEAISCQALPVAVASLSKFLSFYISGKKMPTTEVVVFRTLVT ILTQDIGSETEALNFMLQAQSRASKLGTECFFGLGETGKREQNWFAATCWNLGSRCGKEK KYELCGEFLRLASEFYGYIDTDESGEDKLMICRSIILSVTAMIALEKQTKSALTETQVKL AAELLVRAGKIMSSSLSDGKDCIMEPELIFMYTLLAYDIHGRLNNSAFQLLVVKTFAGSK SCHYNYLLQLGIFASQSPQSNPDVSTFALNECLSALIASASPEYPTIALIIRKLISIASV HKGDTDDEEAILKMYKQAYRIMVGLKEGEYPTEEGKWLAMTAWNRAALPVRLGQFETAKK WLSIGLEIADKVTGMDTYKACMQDYLAGFQTKVSSA
AT4G09140	MLH1	Mlh1(yeast) MLH1(mammals)	ZMM late	Reduction in the formation COs followed by reduction in fertility	Dion et al. 2007; Franklin et al. 2006	> sp|Q9ZRV4|MLH1_ARATH DNA mismatch repair protein MLH1 OS = Arabidopsis thaliana OX = 3702 GN = MLH1 PE = 2 SV = 1 MIDDSSLTAEMEEEESPATTIVPREPPKIQRLEESVVNRIAAGEVIQRPVSAVKELVENS LDADSSSISVVVKDGGLKLIQVSDDGHGIRREDLPILCERHTTSKLTKFEDLFSLSSMGF RGEALASMTYVAHVTVTTITKGQIHGYRVSYRDGVMEHEPKACAAVKGTQIMVENLFYNM IARRKTLQNSADDYGKIVDLLSRMAIHYNNVSFSCRKHGAVKADVHSVVSPSRLDSIRSV YGVSVAKNLMKVEVSSCDSSGCTFDMEGFISNSNYVAKKTILVLFINDRLVECSALKRAI EIVYAATLPKASKPFVYMSINLPREHVDINIHPTKKEVSLLNQEIIIEMIQSEVEVKLRN ANDTRTFQEQKVEYIQSTLTSQKSDSPVSQKPSGQKTQKVPVNKMVRTDSSDPAGRLHAF LQPKPQSLPDKVSSLSVVRSSVRQRRNPKETADLSSVQELIAGVDSCCHPGMLETVRNCT YVGMADDVFALVQYNTHLYLANVVNLSKELMYQQTLRRFAHFNAIQLSDPAPLSELILLA LKEEDLDPGNDTKDDLKERIAEMNTELLKEKAEMLEEYFSVHIDSSANLSRLPVILDQYT PDMDRVPEFLLCLGNDVEWEDEKSCFQGVSAAIGNFYAMHPPLLPNPSGDGIQFYSKRGE SSQEKSDLEGNVDMEDNLDQDLLSDAENAWAQREWSIQHVLFPSMRLFLKPPASMASNGT FVKVASLEKLYKIFERC
AT4G35520	MLH3	Mlh3(yeast) MLH3(mammals)	ZMM late	Reduction in the number of COs, delayed Prophase I	Franklin et al. 2006; Jackson et al. 2006	> sp|F4JN26|MLH3_ARATH DNA mismatch repair protein MLH3 OS = Arabidopsis thaliana OX = 3702 GN = MLH3 PE = 2 SV = 2 MKTIKPLPEGVRHSMRSGIIMFDMARVVEELVFNSLDAGATKVSIFVGVVSCSVKVVDDG SGVSRDDLVLLGERYATSKFHDFTNVETASETFGFRGEALASISDISLLEVRTKAIGRPN GYRKVMKGSKCLHLGIDDDRKDSGTTVTVRDLFYSQPVRRKYMQSSPKKVLESIKKCVFR IALVHSNVSFSVLDIESDEELFQTNPSSSAFSLLMRDAGTEAVNSLCKVNVTDGMLNVSG FECADDWKPTDGQQTGRRNRLQSNPGYILCIACPRRLYEFSFEPSKTHVEFKKWGPVLAF IERITLANWKKDRILELFDGGADILAKGDRQDLIDDKIRLQNGSLFSILHFLDADWPEAM EPAKKKLKRSNDHAPCSSLLFPSADFKQDGDYFSPRKDVWSPECEVELKIQNPKEQGTVA GFESRTDSLLQSRDIEMQTNEDFPQVTDLLETSLVADSKCRKQFLTRCQITTPVNINHDF MKDSDVLNFQFQGLKDELDVSNCIGKHLLRGCSSRVSLTFHEPKLSHVEGYESVVPMIPN EKQSSPRVLETREGGSYCDVYSDKTPDCSLGSSWQDTDWFTPQCSSDRGCVGIGEDFNIT PIDTAEFDSYDEKVGSKKYLSSVNVGSSVTGSFCLSSEWSPMYSTPSATKWESEYQKGCR ILEQSLRLGRMPDPEFCFSAANNIKFDHEVIPEMDCCETGTDSFTAIQNCTQLADKICKS SWGHADDVRIDQYSIRKEKFSYMDGTQNNAGKQRSKRSRSAPPFYREKKRFISLSCKSDT KPKNSDPSEPDDLECLTQPCNASQMHLKCSILDDVSYDHIQETEKRLSSASDLKASAGCR TVHSETQDEDVHEDFSSEEFLDPIKSTTKWRHNCAVSQVPKESHELHGQDGVFDISSGLL HLRSDESLVPESINRHSLEDAKVLQQVDKKYIPIVACGTVAIVDQHAADERIRLEELRTK VLAGKARTVTYLSADQELVLPEMGYQLLQSYSEQIRDWGWICNITVEGSTSFKKNMSIIQ RKPTPITLNAVPCILGVNLSDVDLLEFLQQLADTDGSSTIPPSVLRVLNSKACRGAIMFG DSLLPSECSLIIDGLKQTSLCFQCAHGRPTTVPLVDLKALHKQIAKLSGRQVWHGLQRRE ITLDRAKSRLDNAKS

### Similarity search with HMMER package and phylogenetic inference

HMMER is a more sensitive approach because it employs a whole profile of sequences as a query for similarity searches (Eddy 2011). This way, the program takes advantage of a diversity of amino acids for each position in order to find sequences with a lower level of conservation or more distantly related sequences. This is particularly important for comparisons of large assemblages of lineages of studies of large-scale evolution. In order to build a profile for HMMER searches, one needs to provide an initial trimmed multiple alignment of sequences, (we used MAFFT (Katoh and Standley 2013) and trimAl (Capella-Gutierrez et al. 2009) for alignment and trimming in this pipeline). This initial file is used as input for *hmmbuild* tool in order to generate the profile. The profile is then employed for searches against a database using *hmmsearch* tool. IDs obtained as an output of *hmmsearch* are selected up to an arbitrary threshold (normally e-6) which are used to recover the complete sequences from the database using another tool of the package, the *esl-sfetch* tool. Sequences obtained this way may be used for further analyses, especially phylogeny inference. For phylogenetic inferences, the sequences are aligned and trimmed using the same methods above and directed as input files for a powerful program for phylogeny inference, in this case, IQ-Tree (Minh et al. 2020). The phylogenies obtained this way are then analysed one by one for evolution patterns.

**Fig. 1:**
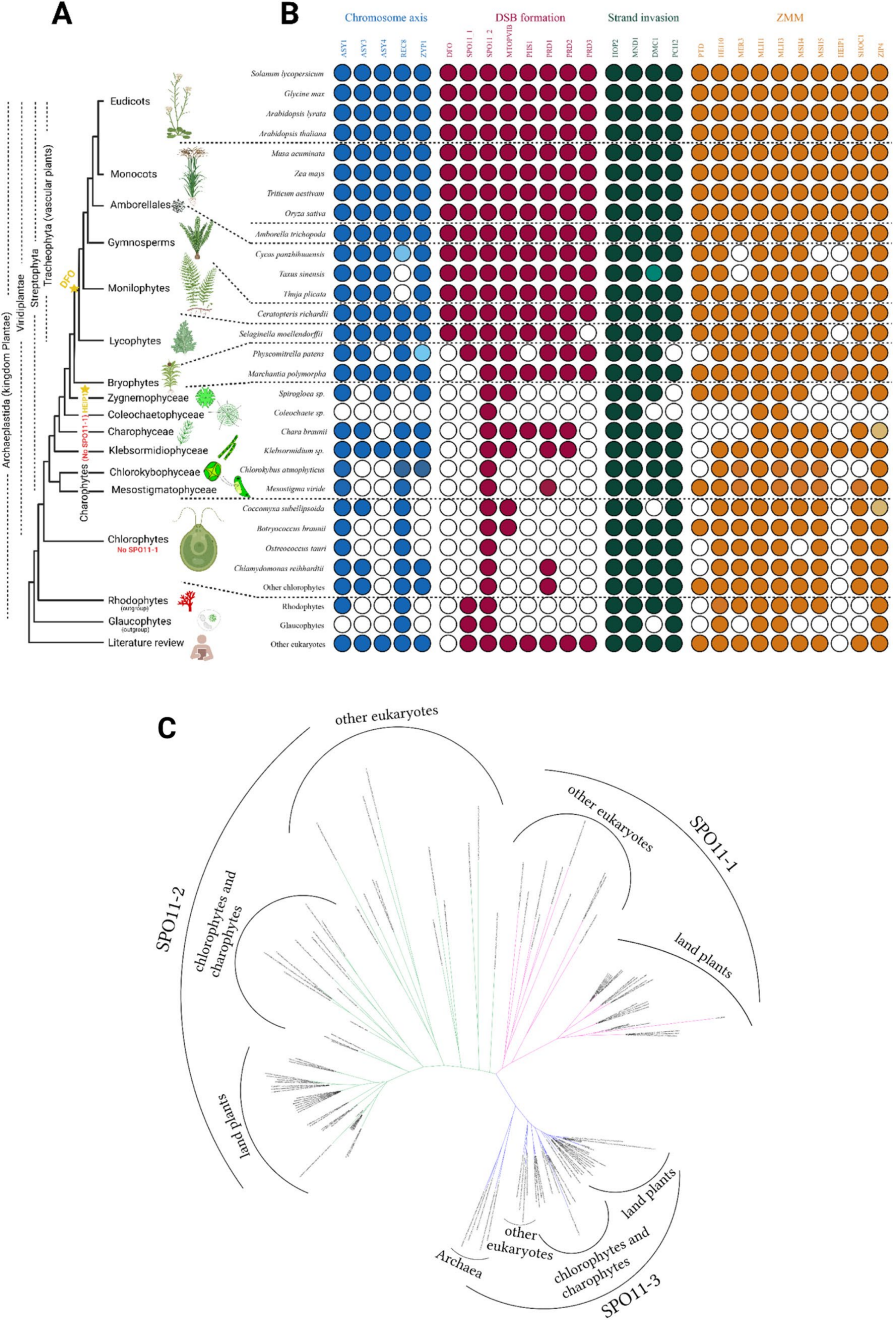
Tracing the conservation of the meiotic machinery among plants. **A** Representative phylogenetic relationship illustration among the main plant lineages, showing the evolutionary events of important meiotic proteins. Loss of *SPO11-1* in Chlorophyta and Charophyta is indicated. Yellow star represents the possible emergence of the meiotic proteins described only in plants till now—HEIP1 and DFO. **B** Using protein homology searches, PSI-BLAST and HMMER, we inferred either presence (coloured circles) or absence (empty circles) of meiotic-specific proteins in all main Viridiplantae lineages. In case of chlorophytes and charophytes, only representative species are shown and the rest are represented as “Others” for chlorophytes. Members of Glaucophyta and Rhodophyta were included in the analysis and represented as outgroups in the figure. See the supplementary table 1 for the whole list of species used in the analysis. Additional information about non-plant homologs obtained based on literature review is added to the figure. Colour code represents the four meiotic pathways according to which the proteins are classified in our analysis. Fully coloured circles = ortholog is detected in our analysis, light coloured circles = a homolog was obtained as a hit but we are unsure whether it is the right ortholog, white coloured (empty) circle = ortholog was not detected. **C** Phylogenetic tree of *SPO11* showing its pattern of duplication across different lineages. Note that the meiotic-specific *SPO11-1* is missing in chlorophytes and charophytes

A comprehensive homology search was carried out by PSI-BLAST and HMMER throughout Archaeplastida. The results from both the analysis were compiled in the final figure. For a simplistic view, in some cases, only few representatives were mentioned for a lineage in the final figure and the rest were concatenated in the “Others” option (Fig. 1A, B). For further details, we recommend the readers to look into the Supplementary Table 2 and the phylogenetic trees (https://data.cyverse.org/dav-anon/iplant/home/gokilavani/Tracing_the_evolution_of_the_plant_meiotic_molecular_machinery). Glaucophytes and rhodophytes were considered to provide a root for your analyses, and as mentioned above, this paper focusses only on discussing the meiotic machinery in Viridiplantae.

## Results and discussion

### Chromosome axis and synaptonemal complex elements are structurally highly conserved but markedly divergent at the sequence level

ASY1, ASY3, REC8 and ZYP1 were detected in all the species or at least in one representative species of all the major Viridiplantae lineages used for the analysis. Exceptionally, we detected ASY4 only in streptophytes, and not in chlorophytes (Fig. 1B). Supporting our analysis, ASY4 was also previously not identified outside land plants (Chambon et al. 2018). On the contrary, ASY3 which interacts with ASY4 (Chambon et al. 2018) was detected in chlorophytes as well. It is important to consider that ASY4 is reported to lack functional domains which constitutes the most conserved region of a protein sequence. Sequence divergence is a feature of the chromosome axis proteins. Axis elements and central elements of the SC exhibit poor similarity between species at the sequence level, but their structure and function are widely conserved (Chambon et al. 2018). The lower sequence conservation could explain why we could not detect *A. thaliana* homolog of ASY4 in distant algal species. For example, *A. thaliana* ASY3, mammalian SYCP2 and yeast Red1 ensures the same function but lacks sequence similarity, likewise *A. thaliana* ASY4 and mammalian SYCP3 (Chambon et al. 2018). Such possibilities cannot be ruled out in this case which is beyond the scope of algorithms used in our analysis.

### The evolution of the meiotic DSB machinery in plants

Among the eight DSB formation proteins we analysed, *DFO* was not detected in Chlorophyta, Charophyta and Bryophyta, *PHS1* and *PRD2* in Chlorophyta and PRD3 and SPO11-1 in Chlorophyta and Charophyta. The rest of the candidates were detected in all Viridiplantae lineages. DFO is a plant-specific protein involved in the formation of DSBs. It has been not reported in other eukaryotic super-groups yet (Zhang et al. 2012). In our analysis, DFO homologs were detected only in the vascular plants and not in other plant lineages, suggesting that *DFO* evolved only in the common ancestor of vascular plants. The homologs of the other three missing candidates PHS1/Rec114, PRD2/Mei4 and PRD3/Mer2 were described to interact with each other and form the RMM complex in *Saccharomyces cerevisiae* (Maleki et al. 2007; Yadav and Claeys Bouuaert 2021). Recently, it has been described, PHS1, PRD2 and the plant-specific DFO forms the RMM-like complex also in *A. thaliana*. PRD3 does not interact with the RMM-like proteins and is proposed to have a different role, likely in coordinating DSB formation and repair mechanisms in *A. thaliana*. PHS1/Rec114 is characterised to have role in DSB formation in species studied so far including maize, except *A. thaliana* where it is proposed not necessary for DSB formation but in regulating meiotic recombination (Vrielynck et al. 2021). Therefore, it becomes evident, and RMM complex has divergent roles in some cases like PRD3 and PHS1. Notably, PHS1/Rec114, PRD2/Mei4, PRD3/Mer2 homologs are conserved across different phyla, but their conservation at the protein sequence level is very weak (Vrielynck et al. 2021). PRD2 and PRD3 have no functional domains reported, except for the presence of several alpha helixes and coiled-coil motifs (De Muyt et al. 2009; Jiang et al. 2009; Vrielynck et al. 2021). The divergence observed among RMM proteins and absence of conserved domains in PRD2, PRD3 explains why we could not detect RMM homologs and plant-specific DFO, part of *A. thaliana* RMM-like complex in distant relatives of our analysis, reconfirming the minimal conservation of RMM proteins.

### SPO11 heterodimerisation has likely evolved in land plants

SPO11 is encoded by a single gene in most organisms (Malik et al. 2007); however, plants differ from yeasts and animals in having several *SPO11* homologs: two paralogs (*SPO11-1* and *SPO11-2*) are involved in meiosis of *A. thaliana* (Grelon et al. 2001; Hartung and Puchta 2001; Hartung et al. 2007; Stacey et al. 2006), where they seem to form a heterodimer that is required for meiotic DSB formation, whereas *SPO11-3* is involved in somatic DNA metabolism (Hartung et al. 2007; Sugimoto-Shirasu et al. 2002; Yin et al. 2002). However, the exact origin of *SPO11-1* and *SPO11-2* duplication and its relation to the heterodimerisation in plants outside *A. thaliana* remained unanswered. This caught our special attention and we further expanded our phylogenetic analysis by including more non-plant representatives from amoeba and archaea. This helped us in tracing the origin of SPO11 duplication in plants. *SPO11-3* (Fig. 1C), which is very similar to archaeal sequences, was detected in all the lineages analysed. Remarkably, among Viridiplantae lineages, our analysis could detect both *SPO11-1* and *SPO11-2* only in land plants, except for *Marchantia polymorpha*, whereas chlorophytes and charophytes have only *SPO11-2* and they seem to lack *SPO11-1* (Fig. 1C). Suggesting two scenarios: 1- heterodimerization of SPO11 evolved in land plants, 2- heterodimerization evolved earlier in eukaryotes but was later lost independently in several lineages and replaced by a homodimer. However, the duplication of *SPO11* is ancestral to eukaryotes, or happened very early in the evolution of eukaryotes as suggested by our phylogenetic analysis and is in agreement as reported earlier (Malik et al. 2007). Members of Amoebozoa, glaucophytes and red algae (grouped under other eukaryotes in Fig. 1C, B), share the same duplication with land plants and have both *SPO11-1* and *SPO11-2* paralogs (Fig. 1C). Thus, we propose that duplication of *SPO11* is ancestral to eukaryotes and most likely *SPO11-1* gene has been lost in both chlorophyte and charophyte lineages after the duplication event. Whether SPO11 activity function as a homodimer in these two lineages needs further investigation.

### Strand invasion is the most conserved meiotic pathway

HOP2, MND1, DMC1, PCH2 are the proteins involved in strand invasion mechanism used for our analysis. It is noteworthy that it is the only group where all the proteins are found in all the lineages in our analysis except some specific cases (Fig. 1B). We observed DMC1 was not detected in glaucophytes analysed but the absence of a complete genome for these species makes it difficult to have a conclusion. DMC1 is the meiotic-specific homolog of bacterial RecA and is required for meiotic homologous recombination. MND1-HOP2 heterodimer promotes DMC1 activity at the DSB sites and promotes stable strand invasion and inter homologue bias (Kerzendorfer et al. 2006). However, some organisms lack DMC1, for example *Drosophila melanogaster, Caenorhabditis elegans, Sordaria macrospora, Neurospora crassa*, which shows that DMC1 can be dispensable. These organisms also lack the accessary factors HOP2 and MND1. However, Viridiplantae and mammals were reported to have DMC1 (Brown and Bishop 2014; Neale and Keeney 2006). Our analysis also shows that all the major Viridiplantae lineages have DMC1 along with HOP2 and MND1 and it may be essential for meiotic homologous recombination in Viridiplantae. PCH2 has a role in chromosome remodelling during SC formation. The initial characterisation of all these proteins in *A. thaliana* revealed their conservation among eukaryotes and observed functional similarity with their non-plant orthologs (Couteau et al. 1999; Kerzendorfer et al. 2006; Lambing et al. 2015; Schommer et al. 2003). Our analysis also concludes the same that strand invasion proteins are the most conserved among the other meiotic proteins we analysed, even at the sequence level. We speculate that such high conservation is linked to their enzymatic function.

### The ZMM pathway is highly conserved and detectable in all plant lineages

PTD, HEI10, MER3, MLH1, MLH3, MSH4, MSH5, SHOC1, ZIP4 are among the ten ZMM pathway proteins analysed, found to be highly conserved in all the major plant lineages. HEIP1 was not detected in chlorophytes. (Fig. 1B). HEIP1 was identified as an interacting partner of HEI10 and suggested to be a member of ZMM pathway as the mutants showed reduced chiasma frequency in rice. It contains a potential plant-specific domain (GCK domain) and not reported outside the plant kingdom till now (Li et al. 2018).This is confirmed in our analysis, and HEIP1 was not detected outside plants and also in the whole chlorophyte lineage. We could not detect HEIP1 in some cases other than chlorophytes as well but at least one species in all other major Viridiplantae lineages had its ortholog. Based on the pattern observed, we propose, HEIP1 is a member of ZMM pathway with possible emergence during the diversification of chlorophytes. PTD orthologs are distant relatives of ERCC1 proteins which are present in both plants and animals (Lu et al. 2014; Wijeratne et al. 2006). SHOC1, the interacting partner of PTD, is a member of XPF superfamily widely present among eukaryotes (Macaisne et al. 2011) and has also been detected in all plant lineages of our study. However, in our analysis, PTD was absent in most of the chlorophytes. PTD may be lost independently from these algae or the protein sequence may be too diverse to be detected by the algorithms given that PTD lacks the conserved motif for endonuclease activity (Wijeratne et al. 2006). Considering both ERCC1 and XPF are structure-specific endonucleases belonging to the XPF superfamily, this difference in the conservation of PTD and SHOC1 implies that individual proteins of the same complex can have different evolutionary trajectories. Another interesting observation is that MER3 was not detected in *Cycas panzhihuaensis* and *Taxus sinensis*. MER3 is highly conserved and *A. thaliana* orthologs were even detected in the most distant algal species used in our analysis. In this case, it may indicate a possible independent loss in the species mentioned above.

## Final remarks

Our comprehensive analysis was able to characterise *SPO11* duplication in plant lineages. SPO11-1 is retained and possibly the heterodimerisation of SPO11-1, and SPO11-2 occurs only in land plants of Viridiplantae. We could also trace the possible origin of the meiotic genes, DFO and HEIP1, which is described only in plants till now. Although there is always a possibility that if the proteins are not detected, it does not necessarily mean they are absent. Notwithstanding the ever-growing volume of genome sequence information, some genomes remain incompletely annotated, which may result in the apparent absence of some proteins in the genome/proteome. Thus, although our results are based on more than one homology search approach, the non-detection of protein homologs in our analysis does not always imply their absence in a given species. Indeed, in a few instances, our failure to detect homologs seems suspicious, for example, the absence of MSH5 in *Cycas panzhihuaensis*, PCH2 in *Physcomitrella patens*, among others. These candidates are highly conserved and detected in all other species analysed. Here it becomes difficult to conclude, whether this is an independent loss scenario or it indicates an artefact. Such cases need more studies to give a concrete answer while other cases discussed had a clear pattern. ASY4, DFO, PHS1, PRD2, PRD3, HEIP1 are absent from all the species of a particular lineage. Here we can be more confident that they are putatively absent or have high sequence divergence to be identified by the algorithms. If meiosis is an ancestral characteristic of eukaryotes, then this raises the question of why some of the proteins in the highly conserved meiotic pathways are putatively absent/not recognised in certain lineages. Possible explanation would be either they are poorly conserved or evolved in some ancestor of the land plants but are absent in the others. If sequence divergence is the case, then it remains to be determined why, within the same pathway, some proteins are more divergent than others; moreover, such an explanation potentially hints at other, yet to identified, evolutionary pressures determining the evolution of these proteins. Most of the meiotic proteins which have enzymatic function or a described functional domain, for example ASY1, SPO11, HEI10, MLH1, MLH3 among others, are observed to be highly conserved in our analysis, whereas proteins like PRD2, PRD3 and ASY4, where functional domains were reported to be absent and do not have an enzymatic function and were less conserved. What also remains to be elucidated is the relevance of lineage-specific loss/gain of certain proteins for meiotic adaptation. Functional validation of selected candidates will be necessary to answer the unanswered questions and to get a complete picture of the different meiotic strategies that have evolved across the massive plant kingdom but we hope our homology search is an attempt to provide first-hand information about the meiotic core proteins across the kingdom.

### Limitations of the study

*Arabidopsis thaliana* protein sequence was used as the initial query in the analysis. We have considered using yeast homologs as the query. Considering, even though meiotic machinery is conserved, not all the proteins are conserved at sequence level between yeast and plants. In some cases, past studies have reported that the yeast and *Arabidopsis* homologs have functional conservation but divergent at the sequence level. The other way around, plant-specific protein like DFO is not reported in yeast. Considering the above points, we narrowed down our aim to look only for the proteins reported in the model plant *Arabidopsis thaliana* among other Viridiplantae lineages and not to look for all the reported meiotic proteins. However, the latter is very exciting but the sequence-based homology search algorithms used in this work will not suffice the needs. Involving structure-based algorithms and carefully looking for functional domains of each protein case by case can be considered but is not the scope of this manuscript.

The sensitivity of the algorithms decreased in the evolutionary distant lineages of *Arabidopsis thaliana* due to sequence divergence and one may think, this could bias our findings. To increase the chances of finding the orthologs, most of the algae which had omics data were included in our analysis. However, we would like to bring to your kind notice that the data sets available for algae were limited. In many cases, the data set available was either vegetative transcriptome or draft genome. This was particularly the case for *Coleochaete* and glaucophytes. Since we are dealing with meiotic-specific candidates, the transcriptome data from vegetative phase may not have their expression, and thus, no hits will be obtained. All the cases, where hits were not obtained, were carefully considered. Due to limitations of the analysis used, no hits do not necessarily mean the protein is absent. Only the cases, where hits were not obtained in the whole lineage was considered as a clear pattern unless specifically mentioned and interpreted further.

#### Author contribution statement

GT and PGH performed the analysis. GT and AM wrote the first draft with subsequent input from PGH and RM. RM and AM conceived and coordinated the study.

## Supplementary Information

Below is the link to the electronic supplementary material.

Supplementary file1 (TXT 3 kb)

Supplementary file2 (XLSX 29 kb)

## Data Availability

All phylogenetic trees and alignments generated in this study can be freely accessed here: https://data.cyverse.org/dav-anon/iplant/home/gokilavani/Tracing_the_evolution_of_the_plant_meiotic_molecular_machinery.

## References

[CR1] Bai X, Peirson BN, Dong F, Xue C, Makaroff CA (1999). Isolation and characterization of SYN1, a RAD21-like gene essential for meiosis in arabidopsis. Plant Cell.

[CR2] Berchowitz LE, Francis KE, Bey AL, Copenhaver GP (2007). The role of AtMUS81 in interference-insensitive crossovers in A. thaliana. PLOS Genet.

[CR3] Bergerat A, de Massy B, Gadelle D, Varoutas P-C, Nicolas A, Forterre P (1997). An atypical topoisomerase II from archaea with implications for meiotic recombination. Nature.

[CR4] Bhatt AM, Lister C, Page T, Fransz P, Findlay K, Jones GH, Dickinson HG, Dean C (1999). The DIF1 gene of Arabidopsis is required for meiotic chromosome segregation and belongs to the REC8/RAD21 cohesin gene family. Plant J.

[CR5] Börner GV, Kleckner N, Hunter N (2004). Crossover/noncrossover differentiation, synaptonemal complex formation, and regulatory surveillance at the Leptotene/Zygotene transition of meiosis. Cell.

[CR6] Brown MS, Bishop DK (2014). DNA strand exchange and RecA homologs in meiosis. Cold Spring Harb Perspect Biol.

[CR7] Cai X, Dong F, Edelmann RE, Makaroff CA (2003). The Arabidopsis SYN1 cohesin protein is required for sister chromatid arm cohesion and homologous chromosome pairing. J Cell Sci.

[CR8] Capella-Gutierrez S, Silla-Martinez JM, Gabaldon T (2009). trimAl: a tool for automated alignment trimming in large-scale phylogenetic analyses. Bioinformatics.

[CR9] Capilla-Perez L, Durand S, Hurel A, Lian Q, Chambon A, Taochy C, Solier V, Grelon M, Mercier R (2021). The synaptonemal complex imposes crossover interference and heterochiasmy in Arabidopsis. Proc Natl Acad Sci USA.

[CR10] Caryl AP, Armstrong SJ, Jones GH, Franklin FCH (2000). A homologue of the yeast HOP1 gene is inactivated in the Arabidopsis meiotic mutant asy1. Chromosoma.

[CR11] Chambon A, West A, Vezon D, Horlow C, De Muyt A, Chelysheva L, Ronceret A, Darbyshire A, Osman K, Heckmann S (2018). Identification of ASYNAPTIC4, a component of the Meiotic chromosome axis. Plant Physiol.

[CR12] Chelysheva L, Diallo S, Vezon D, Gendrot G, Vrielynck N, Belcram K, Rocques N, Marquez-Lema A, Bhatt AM, Horlow C (2005). AtREC8 and AtSCC3 are essential to the monopolar orientation of the kinetochores during meiosis. J Cell Sci.

[CR13] Chelysheva L, Gendrot G, Vezon D, Doutriaux MP, Mercier R, Grelon M (2007). Zip4/Spo22 is required for class I CO formation but not for synapsis completion in Arabidopsis thaliana. PLoS Genet.

[CR14] Chelysheva L, Vezon D, Chambon A, Gendrot G, Pereira L, Lemhemdi A, Vrielynck N, Le Guin S, Novatchkova M, Grelon M (2012). The Arabidopsis HEI10 is a new ZMM protein related to Zip3. PLoS Genet.

[CR15] Chen C, Zhang W, Timofejeva L, Gerardin Y, Ma H (2005). The Arabidopsis ROCK-N-ROLLERS gene encodes a homolog of the yeast ATP-dependent DNA helicase MER3 and is required for normal meiotic crossover formation. Plant J.

[CR16] Corlett RT (2016). Plant diversity in a changing world: status, trends, and conservation needs. Plant Divers.

[CR17] Couteau F, Belzile F, Horlow C, Grandjean O, Vezon D, Doutriaux M-P (1999). Random chromosome segregation without meiotic arrest in both male and female meiocytes of a dmc1 mutant of Arabidopsis. Plant Cell.

[CR18] Crismani W, Portemer V, Froger N, Chelysheva L, Horlow C, Vrielynck N, Mercier R (2013). MCM8 is required for a pathway of meiotic double-strand break repair independent of DMC1 in Arabidopsis thaliana. PLoS Genet.

[CR19] de Massy B, Rocco V, Nicolas A (1995). The nucleotide mapping of DNA double-strand breaks at the CYS3 initiation site of meiotic recombination in Saccharomyces cerevisiae. EMBO J.

[CR20] De Muyt A, Vezon D, Gendrot G, Gallois JL, Stevens R, Grelon M (2007). AtPRD1 is required for meiotic double strand break formation in Arabidopsis thaliana. EMBO J.

[CR21] De Muyt A, Pereira L, Vezon D, Chelysheva L, Gendrot G, Chambon A, Laine-Choinard S, Pelletier G, Mercier R, Nogue F (2009). A high throughput genetic screen identifies new early meiotic recombination functions in Arabidopsis thaliana. PLoS Genet.

[CR22] Dion E, Li L, Jean M, Belzile F (2007). An Arabidopsis MLH1 mutant exhibits reproductive defects and reveals a dual role for this gene in mitotic recombination. Plant J.

[CR23] Dong H, Roeder GS (2000). Organization of the yeast zip1 protein within the central region of the synaptonemal complex. J Cell Biol.

[CR24] Eddy SR (2011). Accelerated profile HMM searches. PLoS Comput Biol.

[CR25] Ferdous M, Higgins JD, Osman K, Lambing C, Roitinger E, Mechtler K, Armstrong SJ, Perry R, Pradillo M, Cunado N (2012). Inter-homolog crossing-over and synapsis in Arabidopsis meiosis are dependent on the chromosome axis protein AtASY3. PLoS Genet.

[CR26] France MG, Enderle J, Rohrig S, Puchta H, Franklin FCH, Higgins JD (2021). ZYP1 is required for obligate cross-over formation and cross-over interference in Arabidopsis. Proc Natl Acad Sci USA.

[CR27] Franklin FCH, Higgins JD, Sanchez-Moran E, Armstrong SJ, Osman KE, Jackson N, Jones GH (2006). Control of meiotic recombination in Arabidopsis: role of the MutL and MutS homologues. Biochem Soc Trans.

[CR28] Frickey T, Lupas A (2004). CLANS: a Java application for visualizing protein families based on pairwise similarity. Bioinformatics.

[CR29] Gerton JL, Hawley RS (2005). Homologous chromosome interactions in meiosis: diversity amidst conservation. Nat Rev Genet.

[CR30] Grelon M, Vezon D, Gendrot G, Pelletier G (2001). AtSPO11-1 is necessary for efficient meiotic recombination in plants. EMBO J.

[CR31] Hartung F, Puchta H (2001). Molecular characterization of homologues of both subunits A (SPO11) and B of the archaebacterial topoisomerase 6 in plants. Gene.

[CR32] Hartung F, Wurz-Wildersinn R, Fuchs J, Schubert I, Suer S, Puchta H (2007). The catalytically active tyrosine residues of both SPO11-1 and SPO11-2 are required for meiotic double-strand break induction in Arabidopsis. Plant Cell.

[CR33] Higgins JD, Armstrong SJ, Franklin FC, Jones GH (2004). The Arabidopsis MutS homolog AtMSH4 functions at an early step in recombination: evidence for two classes of recombination in Arabidopsis. Genes Dev.

[CR34] Higgins JD, Sanchez-Moran E, Armstrong SJ, Jones GH, Franklin FC (2005). The Arabidopsis synaptonemal complex protein ZYP1 is required for chromosome synapsis and normal fidelity of crossing over. Genes Dev.

[CR35] Higgins JD, Buckling EF, Franklin FCH, Jones GH (2008). Expression and functional analysis of AtMUS81 in Arabidopsis meiosis reveals a role in the second pathway of crossing-over. Plant J.

[CR36] Higgins JD, Vignard J, Mercier R, Pugh AG, Franklin FC, Jones GH (2008). AtMSH5 partners AtMSH4 in the class I meiotic crossover pathway in Arabidopsis thaliana, but is not required for synapsis. Plant J.

[CR37] Jackson N, Sanchez-Moran E, Buckling E, Armstrong SJ, Jones GH, Franklin FC (2006). Reduced meiotic crossovers and delayed prophase I progression in AtMLH3-deficient Arabidopsis. EMBO J.

[CR38] Jiang H, Wang FF, Wu YT, Zhou X, Huang XY, Zhu J, Gao JF, Dong RB, Cao KM, Yang ZN (2009). MULTIPOLAR SPINDLE 1 (MPS1), a novel coiled-coil protein of Arabidopsis thaliana, is required for meiotic spindle organization. Plant J.

[CR39] Katoh K, Standley DM (2013). MAFFT multiple sequence alignment software version 7: improvements in performance and usability. Mol Biol Evol.

[CR40] Keeney S, Kleckner N (1995). Covalent protein-DNA complexes at the 5' strand termini of meiosis-specific double-strand breaks in yeast. Proc Natl Acad Sci USA.

[CR41] Keeney S, Giroux CN, Kleckner N (1997). Meiosis-specific DNA double-strand breaks are catalyzed by Spo11, a member of a widely conserved protein family. Cell.

[CR42] Kerzendorfer C, Vignard J, Pedrosa-Harand A, Siwiec T, Akimcheva S, Jolivet S, Sablowski R, Armstrong S, Schweizer D, Mercier R (2006). The Arabidopsis thaliana MND1 homologue plays a key role in meiotic homologous pairing, synapsis and recombination. J Cell Sci.

[CR43] Kuromori T, Azumi Y, Hayakawa S, Kamiya A, Imura Y, Wada T, Shinozaki K (2008). Homologous chromosome pairing is completed in crossover defective atzip4 mutant. Biochem Biophys Res Commun.

[CR44] Lambing C, Osman K, Nuntasoontorn K, West A, Higgins JD, Copenhaver GP, Yang J, Armstrong SJ, Mechtler K, Roitinger E (2015). Arabidopsis PCH2 mediates meiotic chromosome remodeling and maturation of crossovers. PLoS Genet.

[CR45] Li Y, Qin B, Shen Y, Zhang F, Liu C, You H, Du G, Tang D, Cheng Z (2018). HEIP1 regulates crossover formation during meiosis in rice. Proc Natl Acad Sci USA.

[CR46] Liu J, Wu TC, Lichten M (1995). The location and structure of double-strand DNA breaks induced during yeast meiosis: evidence for a covalently linked DNA-protein intermediate. EMBO J.

[CR47] Liu J-G, Yuan L, Brundell E, Björkroth B, Daneholt B, Höög C (1996). Localization of the N-terminus of SCP1 to the central element of the synaptonemal complex and evidence for direct interactions between the N-termini of SCP1 molecules organized head-to-head. Exp Cell Res.

[CR48] Lu X, Liu X, An L, Zhang W, Sun J, Pei H, Meng H, Fan Y, Zhang C (2008). The Arabidopsis MutS homolog AtMSH5 is required for normal meiosis. Cell Res.

[CR49] Lu P, Wijeratne AJ, Wang Z, Copenhaver GP, Ma H (2014). Arabidopsis PTD is required for type I crossover formation and affects recombination frequency in two different chromosomal regions. J Genet Genomics.

[CR50] Macaisne N, Novatchkova M, Peirera L, Vezon D, Jolivet S, Froger N, Chelysheva L, Grelon M, Mercier R (2008). SHOC1, an XPF endonuclease-related protein, is essential for the formation of class I meiotic crossovers. Curr Biol.

[CR51] Macaisne N, Vignard J, Mercier R (2011). SHOC1 and PTD form an XPF-ERCC1-like complex that is required for formation of class I crossovers. J Cell Sci.

[CR52] Maleki S, Neale MJ, Arora C, Henderson KA, Keeney S (2007). Interactions between Mei4, Rec114, and other proteins required for meiotic DNA double-strand break formation in Saccharomyces cerevisiae. Chromosoma.

[CR53] Malik S-B, Ramesh MA, Hulstrand AM, Logsdon JM (2007). Protist homologs of the Meiotic Spo11 gene and Topoisomerase VI reveal an evolutionary history of gene duplication and lineage-specific loss. Mol Biol Evol.

[CR54] Mercier R, Grelon M (2008). Meiosis in plants: ten years of gene discovery. Cytogenet Genome Res.

[CR55] Mercier R, Jolivet S, Vezon D, Huppe E, Chelysheva L, Giovanni M, Nogue F, Doutriaux MP, Horlow C, Grelon M (2005). Two meiotic crossover classes cohabit in Arabidopsis: one is dependent on MER3, whereas the other one is not. Curr Biol.

[CR56] Mercier R, Mezard C, Jenczewski E, Macaisne N, Grelon M (2015). The molecular biology of meiosis in plants. Annu Rev Plant Biol.

[CR57] Meuwissen RL, Offenberg HH, Dietrich AJ, Riesewijk A, van Iersel M, Heyting C (1992). A coiled-coil related protein specific for synapsed regions of meiotic prophase chromosomes. EMBO J.

[CR58] Minh BQ, Schmidt HA, Chernomor O, Schrempf D, Woodhams MD, von Haeseler A, Lanfear R (2020). IQ-TREE 2: new models and efficient methods for phylogenetic inference in the genomic era. Mol Biol Evol.

[CR59] Neale MJ, Keeney S (2006). Clarifying the mechanics of DNA strand exchange in meiotic recombination. Nature.

[CR60] Nguyen LT, Schmidt HA, von Haeseler A, Minh BQ (2015). IQ-TREE: a fast and effective stochastic algorithm for estimating maximum-likelihood phylogenies. Mol Biol Evol.

[CR61] Onn I, Heidinger-Pauli JM, Guacci V, Ünal E, Koshland DE (2008). Sister chromatid cohesion: a simple concept with a complex reality. Annu Rev Cell Dev Biol.

[CR62] Panoli AP, Ravi M, Sebastian J, Nishal B, Reddy TV, Marimuthu MP, Subbiah V, Vijaybhaskar V, Siddiqi I (2006). AtMND1 is required for homologous pairing during meiosis in Arabidopsis. BMC Mol Biol.

[CR63] Peirson BN, Bowling SE, Makaroff CA (1997). A defect in synapsis causes male sterility in a T-DNA-tagged Arabidopsis thaliana mutant. Plant J.

[CR64] Puttick MN, Morris JL, Williams TA, Cox CJ, Edwards D, Kenrick P, Pressel S, Wellman CH, Schneider H, Pisani D (2018). The interrelationships of land plants and the nature of the ancestral embryophyte. Curr Biol.

[CR65] Ronceret A, Doutriaux M-P, Golubovskaya Inna N, Pawlowski Wojciech P (2009). PHS1 regulates meiotic recombination and homologous chromosome pairing by controlling the transport of RAD50 to the nucleus. Proc Natl Acad Sci.

[CR66] Sanchez-Moran E, Santos JL, Jones GH, Franklin FC (2007). ASY1 mediates AtDMC1-dependent interhomolog recombination during meiosis in Arabidopsis. Genes Dev.

[CR67] Sanchez-Moran E, Osman K, Higgins JD, Pradillo M, Cunado N, Jones GH, Franklin FC (2008). ASY1 coordinates early events in the plant meiotic recombination pathway. Cytogenet Genome Res.

[CR68] Schommer C, Beven A, Lawrenson T, Shaw P, Sablowski R (2003). AHP2 is required for bivalent formation and for segregation of homologous chromosomes in Arabidopsis meiosis. Plant J.

[CR69] Stacey NJ, Kuromori T, Azumi Y, Roberts G, Breuer C, Wada T, Maxwell A, Roberts K, Sugimoto-Shirasu K (2006). Arabidopsis SPO11-2 functions with SPO11-1 in meiotic recombination. Plant J.

[CR70] Sugimoto-Shirasu K, Stacey NJ, Corsar J, Roberts K, McCann MC (2002). DNA topoisomerase VI is essential for endoreduplication in arabidopsis. Curr Biol.

[CR71] Sym M, Engebrecht J, Roeder GS (1993). ZIP1 is a synaptonemal complex protein required for meiotic chromosome synapsis. Cell.

[CR72] Tang Y, Yin Z, Zeng Y, Zhang Q, Chen L, He Y, Lu P, Ye D, Zhang X (2017). MTOPVIB interacts with AtPRD1 and plays important roles in formation of meiotic DNA double-strand breaks in Arabidopsis. Sci Rep.

[CR73] Villeneuve AM, Hillers KJ (2001). Whence meiosis?. Cell.

[CR74] Vrielynck N, Chambon A, Vezon D, Pereira L, Chelysheva L, De Muyt A, Mézard C, Mayer C, Grelon M (2016). A DNA topoisomerase VI–like complex initiates meiotic recombination. Science.

[CR75] Vrielynck N, Schneider K, Rodriguez M, Sims J, Chambon A, Hurel A, De Muyt A, Ronceret A, Krsicka O, Mézard C (2021). Conservation and divergence of meiotic DNA double strand break forming mechanisms in Arabidopsis thaliana. Nucleic Acids Res.

[CR76] West AM, Rosenberg SC, Ur SN, Lehmer MK, Ye Q, Hagemann G, Caballero I, Uson I, MacQueen AJ, Herzog F (2019). A conserved filamentous assembly underlies the structure of the meiotic chromosome axis. eLife.

[CR77] Wijeratne AJ, Chen C, Zhang W, Timofejeva L, Ma H (2006). The Arabidopsis thaliana PARTING DANCERS gene encoding a novel protein is required for normal meiotic homologous recombination. Mol Biol Cell.

[CR78] Yadav VK, Claeys Bouuaert C (2021). Mechanism and control of meiotic DNA double-strand break formation in S. cerevisiae. Front Cell Dev Biol.

[CR79] Yin Y, Cheong H, Friedrichsen D, Zhao Y, Hu J, Mora-Garcia S, Chory J (2002). A crucial role for the putative Arabidopsis topoisomerase VI in plant growth and development. Proc Natl Acad Sci.

[CR80] Zhang C, Song Y, Cheng ZH, Wang YX, Zhu J, Ma H, Xu L, Yang ZN (2012). The Arabidopsis thaliana DSB formation (AtDFO) gene is required for meiotic double-strand break formation. Plant J.

[CR81] Zhang L, Kong H, Ma H, Yang J (2018). Phylogenomic detection and functional prediction of genes potentially important for plant meiosis. Gene.

